# *Tetragonia tetragonioides* (Pall.) Kuntze Regulates Androgen Production in a Letrozole-Induced Polycystic Ovary Syndrome Model

**DOI:** 10.3390/molecules23051173

**Published:** 2018-05-14

**Authors:** Bo-Jeong Pyun, Hyun Yang, Eunjin Sohn, Song Yi Yu, Dongoh Lee, Dong Ho Jung, Byoung Seob Ko, Hye Won Lee

**Affiliations:** Herbal Medicine Research Division, Korea Institute of Oriental Medicine (KIOM), Daejeon 34054, Korea; bjpyun@kiom.re.kr (B.-J.P.); hyunyang@kiom.re.kr (H.Y.); ssen4022@kiom.re.kr (E.S.); anyonestar@naver.com (S.Y.Y.); eastdaylight@gmail.com (D.L.); jdh9636@kiom.re.kr (D.H.J.); bsko@kiom.re.kr (B.S.K.)

**Keywords:** *Tetragonia tetragonioides* (Pall.) Kuntze, hyperandrogenism, polycystic ovary syndrome, forskolin, letrozole, steroidogenic enzymes

## Abstract

*Tetragonia tetragonioides* (Pall.) Kuntze (TTK) is a medicinal plant traditionally used to treat various diseases such as diabetic, inflammatory, and female-related disorders. Polycystic ovary syndrome (PCOS) is a common endocrinological disorder in women of reproductive age, and hyperandrogenism is a prominent feature of PCOS resulting in anovulation and infertility. In this study, we investigated the effects of a TTK extract on androgen generation and regulation of steroidogenic enzymes in vitro and in vivo. Human adrenocortical NCI-H295R cells were used to assess the effects of TTK extract on production of dehydroepiandrosterone and testosterone, as well as the protein expression of steroidogenic enzymes. Further, a letrozole-induced PCOS rat model was used in vivo to assess whether dietary administration of TTK extract restores normal hormones and reduces PCOS symptoms. TTK extract significantly inhibited forskolin (FOR)-induced androgen production in NCI-H295R cells and serum luteinizing hormone, testosterone, and follicular cysts, but not estradiol, were reduced in letrozole-induced PCOS rats orally administered the TTK extract. In addition, TTK extract inhibits androgen biosynthesis through the ERK-CREB signaling pathway, which regulates CYP17A1 or HSD3B2 expression. TTK extract could be utilized for the prevention and treatment of hyperandrogenism and other types of PCOS.

## 1. Introduction

Polycystic ovary syndrome (PCOS) is a common and disturbing endocrine disorder that affects approximately 7% of reproductive-aged women and is characterized by hyperandrogenism, ovulatory dysfunction, insulin resistance, polycystic ovarian morphology on ultrasound, weight gain, hirsutism, and other virilizing signs; hyperandrogenism is the main feature of PCOS [1,2,3,4]. According to previous reports, conditions of hyperandrogenic ovaries as well as adrenal androgen secretion appear to be upregulated in PCOS [2,5]. In addition, excess adrenal androgen levels, especially elevated levels of adrenal androgen metabolites, including dehydroepiandrosterone (DHEA) and dehydroepiandrosterone sulfate (DHEAS), have been reported in ~50% of PCOS patients [5,6,7,8]. Generally, androgen production is necessary for estrogen synthesis, which occurs in all healthy women; hyperandrogenic conditions are characterized by dysfunctional production of androgens or improper conversion to estrogens [9]. The high levels of luteinizing hormone (LH) observed in common PCOS patients is related to the mechanisms of hyperandrogenism, including exposure of the ovarian theca and granulosa cells to LH and increased levels of cAMP. Further, stimulation of steroidogenic enzymes leads to the conversion of cholesterol to steroids hormones [1,10].

Previous studies suggest that the symptoms of PCOS are induced by letrozole, a nonsteroidal aromatase inhibitor that blocks the conversion of androgens to estrogen by inhibiting the aromatase enzyme [11,12]. Letrozole treatment in adult rats for at least 21 consecutive day results in failure of the ovarian cycle [11,12,13] or an irregular estrous cycle [13]; in addition, the number of follicular cysts is increased in the ovaries, with fewer or no corpus lutea [13]. Under polycystic conditions, follicular atresia, thin granulosa cell layers, and thickened theca cell layers are observed in the ovaries [13,14]. Endocrine imbalances include elevated levels of LH and testosterone, which reflects the accumulation of endogenous androgen secretion attributable to blockade of aromatase activity in the ovaries [13,15]. Many features of human PCOS are observed among various rodent models, including the letrozole-induced rat model [11,13,14,16,17].

Important proteins in the androgen biosynthetic pathway include 17α-hydroxylase/17,20-lyase (CY17A1), the 3β-hydroxysteroid dehydrogenase/Δ^5^-Δ^4^-isomerase type 2 (HSDB2) enzyme, and DHEA, which are steroidogenic regulatory proteins that regulate cholesterol transport and convert steroids to adrenal androgens [1,18]. Expression of these enzymes is increased in ovarian theca cells from PCOS patients, attributable to androgen excess [19,20,21]. To date, the pathomechanisms of PCOS in the regulation of adrenal androgen production have not been fully explored. Our study focused, in part, on the steroidogenic pathway to determine the effects of adrenal androgens in androgen biosynthesis and to facilitate close examination of steroidogenic enzymes using the NCI-H295R steroidogenic cell line under indirect androgen excess conditions of PCOS. According to several reports, insulin resistance is a major pathologic feature in women with hyperandrogenic PCOS. Metformin and pioglitazone are widely used to treat insulin resistance and to regulate steroidogenic enzymes such as HSD3B2 and CYP17A1 [22,23,24,25,26,27]; therefore, they were used here as positive controls in our PCOS-like model.

*Tetragonia tetragonioides* (Pall.) Kuntze (TTK) is an edible halophyte belonging to the Aizoaceae family that is also known as New Zealand spinach, sea spinach, and Botany Bay spinach. This plant is widespread from Korea, China, Japan, Argentina, Chile, New Zealand and throughout Australia [28,29,30]. It also can be consumed as a salad or herb in the West and is well known as a beneficial traditional herbal medicine for treating stomach diseases such as stomach ulcers and gastritis [29,31,32]. Previous studies of the antioxidant, antidiabetic, anti-inflammatory, and life prolongation effects of TTK crude extracts and fractions have been published [33,34,35]. Furthermore, the major constituents of TTK have been isolated and include soluble polysaccharides, sphingosine, diterpenoids, flavonol glycosides, and lignan amides such as cerebrosides, methyl linoleate, methyl coumarate, methyl ferulate, (2*S*)-1-*O*-stearoyl-3-*O*-β-d-galactopyranosyl-sn-glycerol, 1-*O*-caffeoyl-β-d-glucopyranoside, N-*trans* caffeoyltyramine, cannabisin B, and cannabisin A [31,32,36,37,38]. As we reported previously, TTK extract decreases proinflammatory cytokines and protects estrogen-deficient rats against disturbances in energy and glucose metabolism [39]. Additionally, TTK extract has been used to treat inflammatory diseases and to improve health in women. 

In the present study, we demonstrate that TTK extract inhibits serum testosterone and LH, as well as follicular cyst development in a letrozole-induced PCOS-like rat model. Furthermore, we show that TTK extract protects against hyperandrogenism. The underlying mechanisms are related to regulation of androgen biosynthesis through the extracellular signal-related kinase and cAMP response element-binding protein (ERK-CREB) pathway, which is involved in forskolin (FOR)-induced androgen production in human adrenal NCI-H295R cells.

## 2. Results

### 2.1. Quantitative Analysis of Marker Compounds in TTK Extract

**Figure 1:**
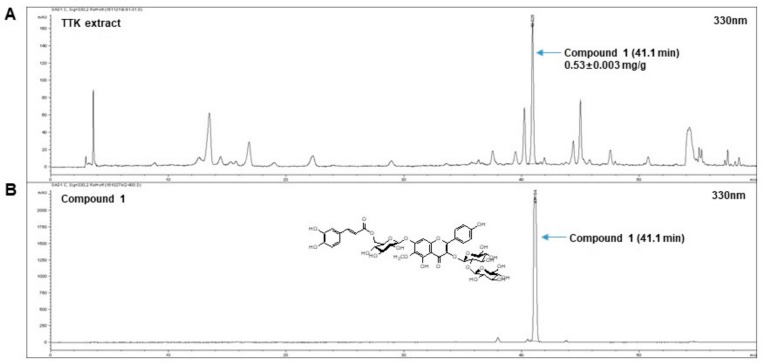
Fingerprinting analysis of (**A**) *Tetragonia tetragonioides* Kuntze (TTK) extracts and (**B**) the marker compound. For fingerprinting analysis, TTK extracts and the marker reference compound were subjected to high-performance liquid chromatography (HPLC).

Using a stock solution of compound **1**, regression equations were measured for six concentrations. Linearity was tested from the correlation coefficient (R^2^) of the calibration curves. The calibration curve for compound 1 (y = 167.07x + 45.007 and 0.999[R^2^]) was within the tested range. The contents of compound 1 in the 70% ethanol TTK extract were 0.53 ± 0.003 mg/g in triplicate at the retention time of 41.1 min (Figure 1).

### 2.2. Cytotoxicity on NCI-H295R Cells of FOR and TTK Extract

**Figure 2:**
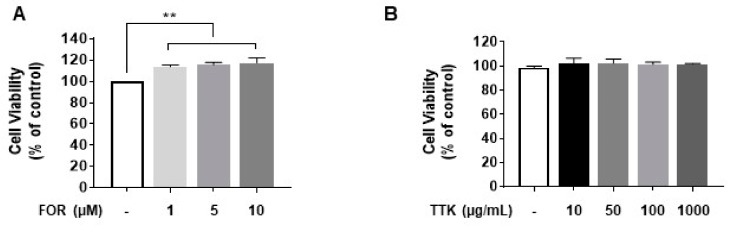
Effects of forskolin (FOR) and *Tetragonia tetragonioides* Kuntze (TTK) extract on viability in serum-deprived NCI-H295R cells. (**A**,**B**) NCI-H295R cells were incubated in the presence of the indicated concentrations of FOR or TTK extract for 24 h. Cell viability was determined using the 3-(4,5-dimethylthiazol-2-yl)-2,5-diphenyltetrazolium bromide (MTT) assay, and absorbance was measured at 540 nm. Data are representative of three independent experiments and expressed as the means ± SD ** *p* < 0.01 vs. control.

MTT assays were performed to investigate the cytotoxicity of FOR in our system. NCI-H295R cells were treated with various concentrations (1–10 μM) FOR for 24 h. As shown in Figure 2A, cell viability was higher in FOR-treated cells than that in the control cells. Next, to determine non-toxic concentrations of the TTK ethanol extract, we examined cell proliferation. As shown in Figure 2B, the average cell viability in TTK-extract treated cells was more than 90% for the various concentrations (10–1000 μg/mL).

### 2.3. TTK Extract Inhibits FOR-Induced DHEA and Testosterone Production in NCI-H295R Cells

**Figure 3:**
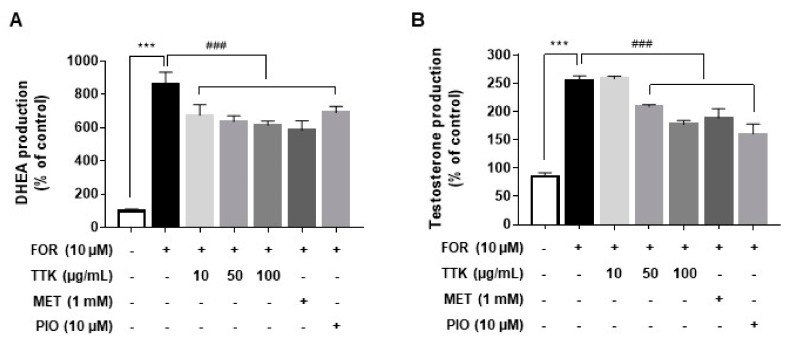
Effects of *Tetragonia tetragonioides* Kuntze (TTK) extract on forskolin (FOR)-induced dehydroepiandrosterone (DHEA) and testosterone production in serum-deprived NCI-H295R cells. (**A**,**B**) Cells were pretreated with TTK extract (10, 20, 50, and 100 μg/mL) for 30 min prior to exposure to 10 μM FOR for 24 h. DHEA or testosterone was measured in cell culture medium with a competitive enzyme-linked immunospecific assay following the manufacturers’ recommendations. Metformin (MET, 1 mM) and pioglitazone (PIO, 10 μM) were used as positive controls for the inhibitory effects of FOR. Data are representative of three independent experiments and expressed as the means ± SD; *** *p* < 0.001 vs. control; ^###^ *p* < 0.001 vs. FOR. MET, metformin; PIO, pioglitazone.

We examined the effects of TTK extract on DHEA or testosterone production in the NCI-H295R cell line, which is an indirectly established in vitro model of the androgen overproduction such as observed in PCOS. Similar to the conditions observed for PCOS, elevated androgen levels are present in these cells. Therefore, we performed experiments in the presence of FOR, which regulates the effects of higher levels of DHEA or testosterone. Androgens, DHEA, and testosterone were analyzed in conditioned culture medium. Under FOR-stimulated conditions in NCI-H295R cells, TTK extract reduced DHEA or testosterone concentrations in a dose-dependent manner (Figure 3A,B). Similar effects were observed for metformin and pioglitazone.

### 2.4. TTK Extract Inhibits Folliculogenesis in Letrozole-Induced PCOS Rats

**Figure 4:**
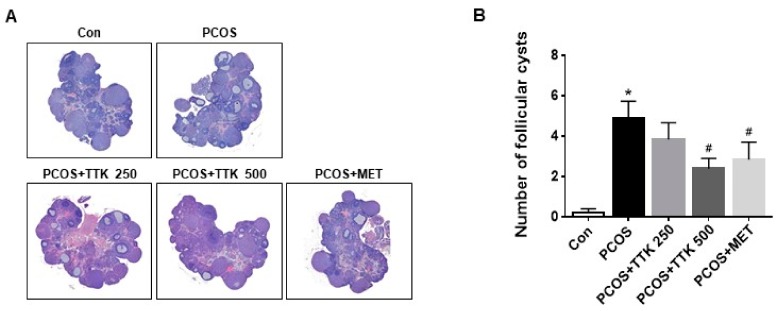
Effects of *Tetragonia tetragonioides* Kuntze (TTK) extract on letrozole-induced rat ovary histopathology. (**A**) Ovary sections (stained with hematoxylin and eosin) are shown, and (**B**) the number of follicular cysts is depicted. Con, vehicle control; PCOS, polycystic ovary syndrome induced by letrozole only; PCOS+TTK250 (letrozole induction + TTK (250 mg/kg/body weight [BW]); PCOS+TTK500, letrozole induction + TTK (500 mg/kg/BW)); PCOS+MET, letrozole induction + metformin (500 mg/kg/BW). * *p* < 0.05 compared with the vehicle group (Con); ^#^ *p* < 0.05 compared with the PCOS group.

Polycystic ovary (PCO) is a serious condition that can lead to pelvic pain, infertility, obesity, and metabolic disorders [16,17,40]. In the present study, we used letrozole-induced rats to mimic PCOS-like conditions and assess the effects of TTK extract in female rats. The effect of (TTK) extract on ovarian tissue morphology in letrozole-induced PCOS rats is presented in Figure 4. Ovarian tissues were stained with H&E. Classical PCOS morphology includes ovarian cortical thickening, multiple tiny capsular follicular cysts, hyperplasia, luteinized inner theca, and other signs, which indicate that folliculogenesis has ceased. Ovary size did not change in any experimental groups; however, a greater number of cystic follicles was observed in the letrozole-induced PCOS group. In the positive control group, the number of cystic follicles was lower in metformin-treated (500 mg/kg/BW) PCOS rats than that in untreated PCOS rats. Additionally, the high number of cystic follicles was reduced by treatment with a higher dose of TTK extract (500 mg/kg/BW), but not lower doses of TTK extract.

### 2.5. TTK Extract Inhibits Serum LH, Testosterone, and E2 Level(s) in Letrozole-Induced PCOS Rats

**Figure 5:**
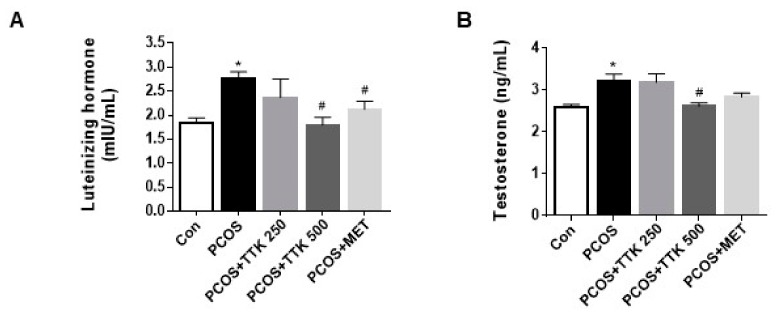

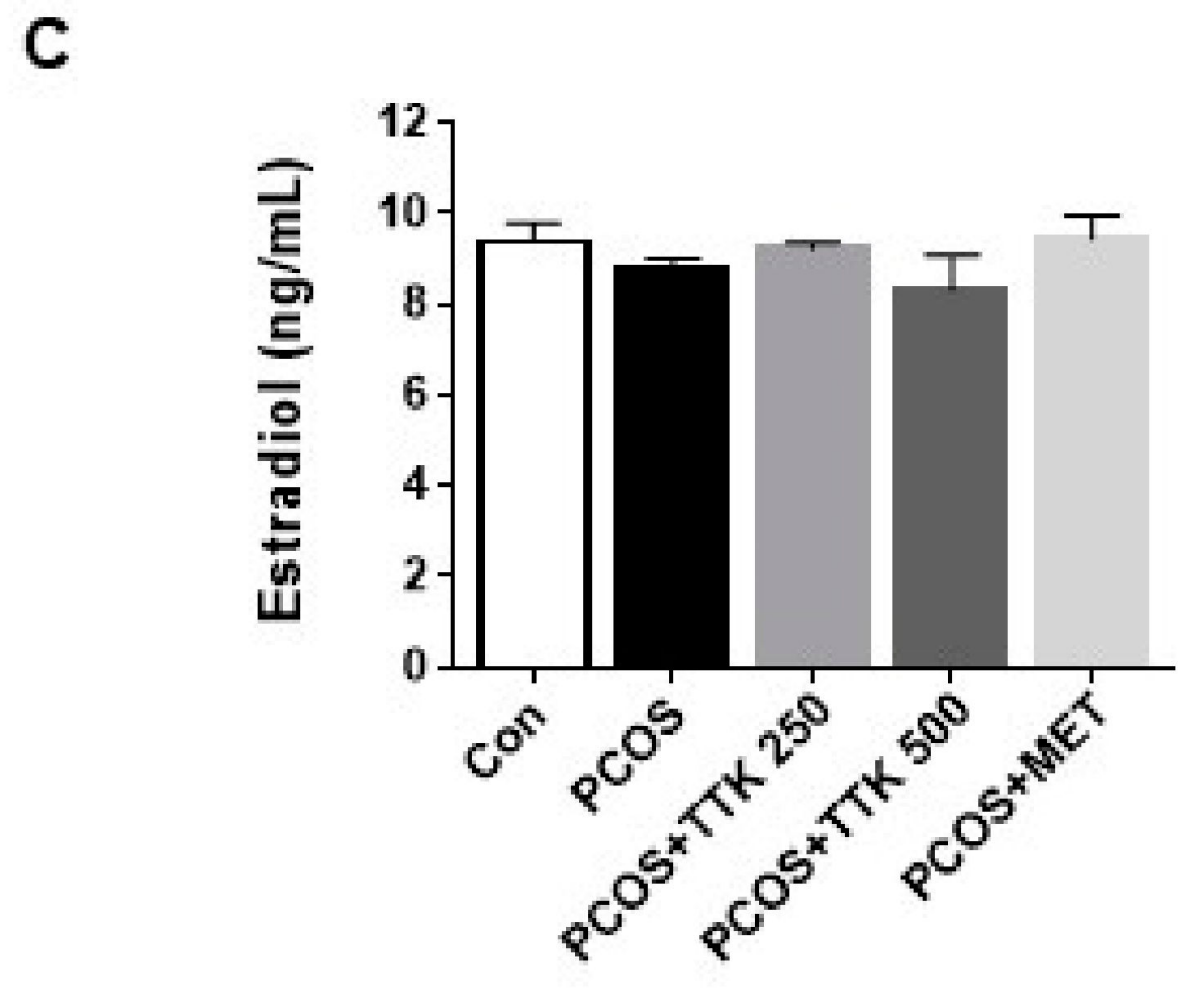
Comparison of the mean levels of luteinizing hormone, testosterone, and estradiol in the different treatment groups. The rats were divided into five groups (Con, PCOS, PCOS + TTK250, PCOS + TTK500, and PCOS + MET). The serum levels of (**A**) luteinizing hormone, (**B**) testosterone, and (**C**) estrogen were assessed by enzyme-linked immunosorbent assay. * *p* < 0.05 compared with the vehicle group (Con); ^#^ *p* < 0.05 compared with the PCOS group. Con, control; PCOS, polycystic ovary syndrome; MET, metformin.

Differences in body weight and daily food intake between rats fed the standard diet in the presence or absence of letrozole for 4 weeks were evaluated. Rats from the letrozole-induced groups had similar body weight gains and food intake; however, weight gain and food intake in the positive control and TTK-extract treated groups were not significantly different from those in the PCOS group (data not shown). As shown in Figure 5, letrozole-induced PCOS resulted in significantly higher levels of LH and testosterone in the PCOS group; however, serum levels of E2 did not fluctuate among groups. In the positive control group, LH levels were significantly lower in metformin-treated (500 mg/kg/BW) rats than those in untreated PCOS rats; however, serum testosterone levels were slightly, but insignificantly changed. In addition, the elevated serum levels of LH and testosterone were significantly decreased by higher doses of TTK extract (500 mg/kg/BW), but not a lower dose (TTK; 250 mg/kg/BW).

### 2.6. TTK Extract-Mediated Inhibition of FOR-Induced DHEA or Testosterone Production in NCI-H295R Cells Involves CYP17A1 and HSD3B2 Enzymes via the ERK-CREB Signaling Pathways

**Figure 6:**
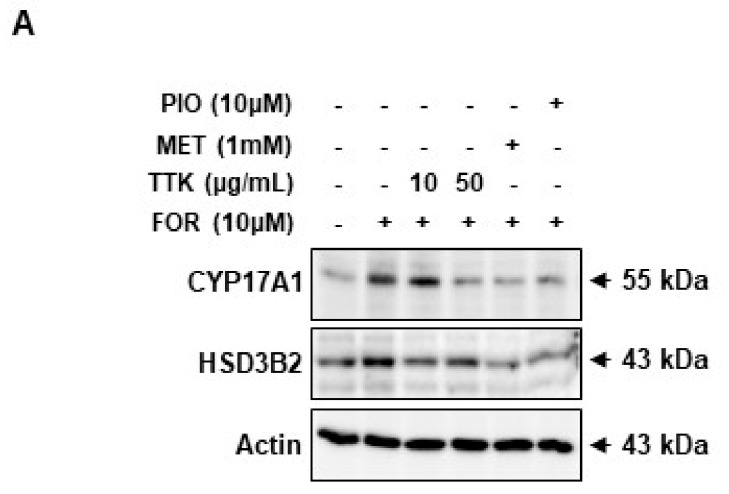

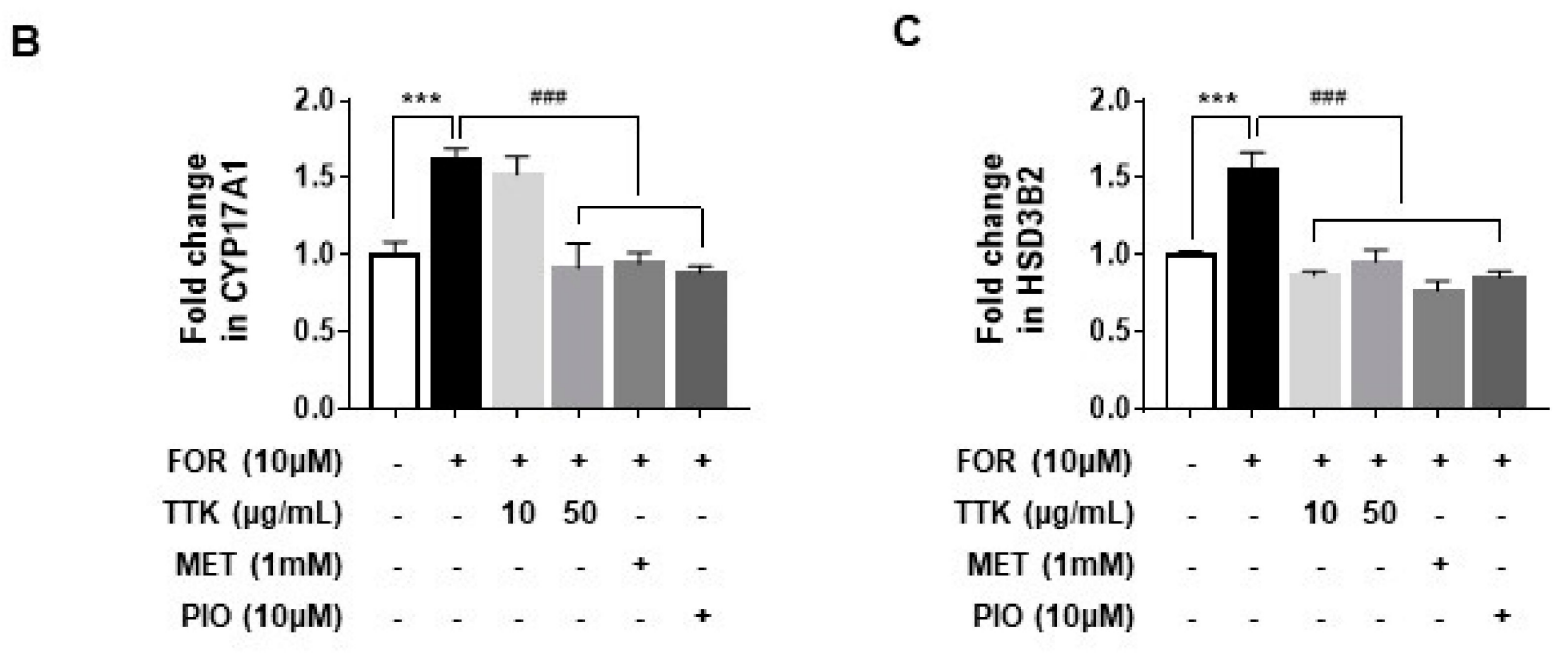
Effects of *Tetragonia tetragonioides* Kuntze (TTK) extract on CYP17A1 and HSD3B2 in serum-deprived NCI-H295R cells. (**A**–**C**) Total cell lysates were analyzed via western blots using specific antibodies for CYP17A1 and HSD3B2. Total actin served as a loading control. Data are representative of three independent experiments and are expressed as the means ± SD; *** *p* < 0.001 vs. control; ^###^ *p* < 0.001 vs. FOR. MET, metformin; PIO, pioglitazone.

**Figure 7:**
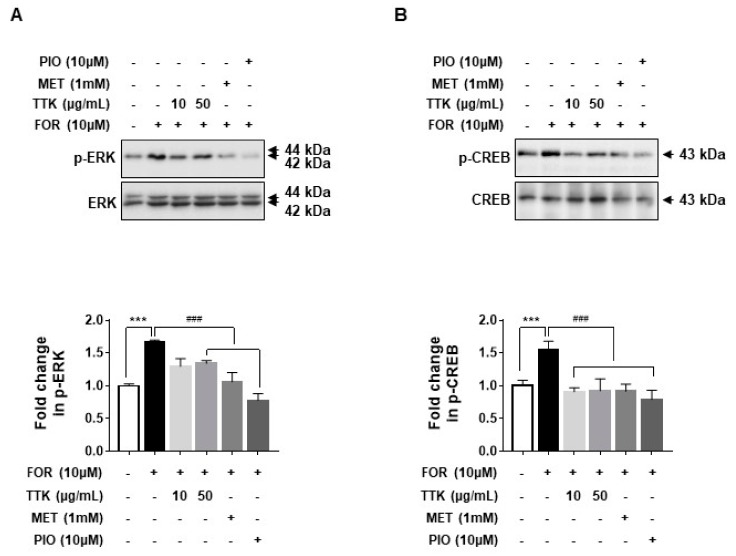
Effects of *Tetragonia tetragonioides* Kuntze (TTK) extract on extracellular signal-related kinase (ERK) and cAMP response element binding protein (CREB) in serum-deprived NCI-H295R cells. (**A**,**B**) Total cell lysates were analyzed by western blots using specific antibodies for phosphorylated ERK and CREB. Total ERK and CREB served as loading controls. Data are representative of three independent experiments and expressed as the means ± SD; *** *p* < 0.001 vs. control; ^###^ *p* < 0.001 vs. FOR. MET, metformin; PIO, pioglitazone.

As shown in Figure 6A,B, elevated protein expression of the androgen synthesis enzymes, CYP17A1 and HSD3B2, was observed in NCI-H295R cells exposed to FOR. Increased CYP17A1 and HSD3B2 protein levels implicate activation of the ERK-CREB signaling pathways; therefore, we treated NCI-H295R cells with FOR in the presence or absence of TTK extract and then measured CYP17A1 and HSD3B2 protein expression; ERK and CREB phosphorylation was subsequently measured. Pre-treatment with TTK extract slightly decreased FOR-induced phosphorylation of ERK and CREB (Figure 7A,B). Taken together, these results show that FOR-induced DHEA or testosterone production in NCI-H295R cells might be partially mediated by activation of the ERK and CREB signaling pathways.

## 3. Discussion

The results of this study show that TTK extract is an effective inhibitor of androgen biosynthesis, which results from steroidogenic enzymes and ERK-CREB signaling. PCOS is a complex medical condition that develops in fertile women and is characterized by oligo-ovulation, anovulation, excessive androgens, polycystic ovarian morphology on ultrasound, and several other disorders [41,42]. Because hyperandrogenism is a major pathophysiological feature of PCOS, we examined the excessive androgen generation resulting from hormone imbalances and CYP17A1 and HSD3B2 activity—key enzymes involved in steroidogenesis. According to previous studies, FOR increases levels of cAMP in cells and elevates the level of other hormones, including androgens such as testosterone [43,44]. In addition, metformin significantly inhibits androgen production in FOR-stimulated ovarian cells [44], and pioglitazone inhibits androgen production in NCI-H295R cells by regulating CYP17 and HSD3B2 [27].

Cell viability increased in cells treated with various concentration of FOR, indicating that TTK extract, metformin, and pioglitazone significantly inhibit FOR-induced effects on NCI-H295R cell viability (Figure 2). Survival in NCI-H295R cells is closely associated with the development of PCOS, attributable to hormonal imbalances. We previously demonstrated that TTK extract protects estrogen-deficient rats against disturbances in energy and glucose metabolism and decreases pro-inflammatory cytokines. In this study, we confirm the potent efficacy of TTK extract in women experiencing menopausal symptoms attributable to a hormone imbalance. In this regard, the present data indicate that TTK extract provides protection against hormone-related diseases, including PCOS. 

In this study, FOR was identified as a stimulator of androgen, which was augmented by DHEA or testosterone secretion in NCI-H295R cells; DHEA or testosterone levels were decreased by treatment with TTK extract, metformin, or pioglitazone (Figure 3). Specifically, we exposed female rats to letrozole (an aromatase inhibitor), which induces conditions similar to those observed for PCOS. We then examined LH, testosterone, and E2 levels in the serum and evaluated histopathological changes attributable to treatment with TTK extract in female rats with letrozole-induced PCOS-like condition. Elevated serum LH and testosterone levels are related to endocrine imbalances and contribute to PCOS symptoms such as ovarian dysfunction and irregular ovarian or estrous cycles [45]. In our rat model, we observed fewer follicular cysts and lower serum LH and testosterone levels in letrozole-induced rats treated with 500 mg/kg/BW TTK extract than those in untreated letrozole-induced rats (Figure 4 and Figure 5). In previous studies, letrozole was found to cause imbalances in ovarian function and hormones, including hypersecretion of LH and androgens [13,14,41]. Ghafurniyan et al., showed that multiple cysts were observed, and LH and testosterone levels were effectively reduced by treatment with an herbal extract in a PCOS rodent model [46]. Moreover, herbal extracts have been demonstrated to be effective for improving the symptoms of PCOS [47,48,49]. Previously, the consumption of phytoestrogen components led to reducing LH secretion [50], and reduction of LH secretion is mediated via estrogen receptor 1 (ESR1) [51]. ESR1 is known to be involved in the regulation of the negative feedback of estrogen on LH secretion in ESR1-/- mice [52,53]. These findings suggest that our experimental model closely represents the typical symptoms of the imbalance of ovarian hormone, and the TTK herbal extract may be useful as an adjunctive therapy via estrogenic effect or estrogen receptor agonist for the imbalance of ovarian hormone in letrozole-induced PCOS model.

The physiological regulation of androgens is mediated by LH and adrenocorticotropic hormone (ACTH), which promote the activity of steroidogenic enzymes via the second messenger cAMP, ultimately increasing androgen biosynthesis [1,54]. Our data suggest that FOR-induced production of DHEA or testosterone was significantly reduced by TTK extract; FOR is a natural activator of cAMP. We also demonstrate that TTK extract suppresses FOR-induced androgen generation through CYP17A1 and HSD3B2 in the androgenic pathway. Our results show that FOR-induced androgen production in NCI-H295R cells is associated with phosphorylation of ERK and CREB. These changes were repressed by treatment with TTK extract; therefore, we suggest that it plays a key role in androgenic PCOS conditions. Further study will be required to elucidate the evident mechanisms underlying the decrease in CYP17A1 and HSD2B2 and to determine how the ERK-CREB pathway is related to such decrease. In particular, the HSD3B2 enzyme acts as a key enzyme in the synthesis of cortisol and progesterone/aldosterone [55,56]. In this study, effects of the TTK extract were examined under conditions of limited androgens. Therefore, future studies are needed to investigate the activity of TTK extract on hormone production, including cortisol and aldosterone, and other steroidogenesis pathways involving HSD3B2 enzyme.

## 4. Materials and Methods

### 4.1. Plant Material

*Tetragonia tetragonioides* (Pall.) Kuntze (TTK) was purchased from an Oriental medicine company in Kwangmyung-Dang (Ulsan, Korea). The plants were authenticated by Dr. Byoung Seob Ko at the Korea Institute of Oriental Medicine (KIOM) in Daejeon, Korea; the voucher specimen (KIOM M 130081-3) was deposited in the Herbal Medicine Research Division of KIOM.

### 4.2. Preparation and Fingerprinting Analysis of TTK Extract

Dried TTK (4 kg) was extracted with 70% ethanol (40 L) for 3 days at 25–30 °C and then filtered. After concentrating the 70% ethanol layers and lyophilization, the TTK extracts were stored at −70 °C until use. Final yield of the 70% ethanol extract was 22.43% *w*/*w* (992.6 g). A quantitative analysis was performed using an 1100 series high-performance liquid chromatographδ system (HPLC, Agilent Technologies, Santa Clara, CA, USA). The analytical column with an Atlantis C18 (4.6 × 250 nm, 5 μm, Waters, MA, USA) was maintained at 30 °C during the experiment. The mobile phase included distilled water (DW) with 0.1% trifluoroacetic acid (A) and acetonitrile (B). The gradient flow was as follows: 0–25 min, 10–15% (*v*/*v*) B; 25–50 min, 15–30% (*v*/*v*) B; 50–60 min, and 30–100% (*v*/*v*) B. The analytes were detected at 330 nm and operated at a mobile phase flow rate of 1.0 mL/min. The injection volume was 10 μL. The data were acquired and processed by ChemStation software (Agilent Technologies). We isolated marker compound **1** from TTK, and the compound were identified as 6-methoxykaempferol-3-*O*-β-d-glucosyl(1′′′→2″)-β-D-gluco-pyranosyl-(6″″-(*E*)-caffeoyl)-7-*O*-β-d-glucopyranoside via nuclear magnetic resonance (NMR) spectroscopy and mass spectrometry (MS) experiments.

### 4.3. Cell Culture and Reagents

Human adrenocortical NCI-H295R (NCI-H295R) cells were obtained from American Type Culture Collection (ATCC-LGC Standards GmbH, Wesel, Germany). Cells were cultured under standard conditions in Dulbecco's modified Eagle's/Ham's F-12 medium (DMEM/F12; Gibco, Life Technologies Europe BV, Bleiswijk, The Netherlands) supplemented with 2.5% Nu-serum (BD Biosciences, Breda, The Netherlands), 1% insulin/transferrin/selenium (ITS, BD Biosciences), and 1% penicillin and streptomycin (pen/strep, Gibco, Life Technologies Europe BV). Other biochemical reagents, including metformin and pioglitazone, were obtained from Sigma-Aldrich (St. Louis, MO, USA) unless otherwise specified.

### 4.4. Cell Proliferation

NCI-H295R cells were seeded onto 96-well plates at a density of 1 × 10^3^ cells/well and incubated under serum-free conditions prior to treatment with foskolin (FOR) and TTK extract. Cell viability was evaluated by the 3-(4,5-dimethylthiazol-2-yl)-2,5-diphenyltetrazolium bromide (MTT) assay. After treatment, MTT solution (0.5 mg/mL) was added to each well and incubated for 4 h at 37 °C. The supernatant was removed, and the obtained formazan product was dissolved in 100 μL dimethyl sulfoxide (DMSO) with stirring for 15 min on a shaker; absorbance was measured with a microtiter plate reader (BIO-TEK, Synergy HT, Winooski, VT, USA) at 570 nm. The percentage of viable cells in each treatment group was determined using control experimental optical density (OD) values.

### 4.5. DHEA and Testosterone Measurements

DHEA and testosterone concentrations were measured using a competitive enzyme-linked immunosorbent assay (ELISA) kit (DHEA, catalog no. ADI-900–093, Enzo Life Sciences, Sigford Road, Exeter, UK; testosterone, catalog no. 582701, Cayman Chemical, Ann Arbor, MI, USA) following the manufacturers’ protocols. NCI-H295R cells were plated onto 96-well plates at 1 × 10^3^ cells/well and incubated under serum-free conditions before exposure to FOR (10 μM) in the presence or absence of TTK extract, metformin, or pioglitazone for 24 h. DHEA or testosterone released into the media was measured in triplicate against standards made up in medium using an ELISA kit. The results were normalized to the controls.

### 4.6. Experimental Animals and Treatments

Female Wistar rats (6 weeks old, weighing 120–140 g; total n = 30, n = 6 per group) were purchased from Dahan Biolink (Eumseong, South Korea) and adapted to laboratory conditions (temperature: 20 ± 2 °C, relative humidity: 45 ± 5%, light/dark cycle: 12 h) for 1 week. Rats were fed a standard rodent chow diet (Nestle Purina, St. Louis, MO, USA) and were euthanized with an intraperitoneal injection of Zoletil:Rompun (3:1) 24 h after the last treatment. Letrozole (Tokyo Chemical Industry Co., Ltd., Tokyo, Japan) dissolved in 0.5% carboxymethyl cellulose (CMC, Tokyo Chemical Industry Co., Ltd.) was used to induce polycystic ovaries for 3 weeks in female Wistar rats. To examine the effects of TTK extract on polycystic ovaries, the rats were orally administered 500 mg/kg/body weight (BW) metformin (Tokyo Chemical Industry Co., Ltd.), 250 or 500 mg/kg/BW TTK extract, or 0.1% CMC as a vehicle control daily for 4 weeks. Dosages were adjusted according to changes in body weight. All animal experimental procedures were approved by the Ethics Committee of Korea Institute of Oriental Medicine (approval No. 16-024).

### 4.7. Histopathological Analysis

The ovaries were fixed by inflating the tissue with 10% neutral buffered formalin. The tissues were embedded in paraffin, cut into sections (5 microns), and stained with hematoxylin and eosin (H&E; Sigma-Aldrich, MO, USA). All tissue samples were examined, photographed, and scored in a blinded fashion under a light microscope (BX43; Olympus, Tokyo, Japan). Images were captured using an Olympus DP-73 (Olympus) controller and cellSens standard (Olympus) under a microscope. The number of follicular cysts was counted under a microscope.

### 4.8. Serum Hormone Analysis

Blood samples were collected directly from the inferior vena cava using a 1-mL syringe at the end of the experiment. Serum was obtained by centrifugation at 2000× *g* for 10 min and stored at −70 °C until use. Serum LH levels were measured using a rat LH ELISA kit (Cusabio Biotech, Wuhan, China). Serum testosterone levels were measured using a testosterone ELISA kit (Abcam, Cambridge, UK). 17β-Estradiol (E2) levels were measured using an estradiol (rat) ELISA kit (BioVision, Mountain View, CA, USA). All kits were used according to the manufacturers’ instructions.

### 4.9. Determination of Protein Levels

NCI-H295R cells were plated at 2 × 10^5^ cells/dish in 60-mm culture dishes 24 h before drug treatment. Cells were then treated with 10 μM FOR in the presence or absence of TTK extract, metformin, or pioglitazone for 24 h. Cells were lysed with Laemmli sample buffer (Bio-Rad, Hercules, CA, USA), heated at 100 °C for 5 min, and electrophoresed with 25~30 µg protein/lane on denaturing sodium dodecyl sulfate-polyacrylamide (SDS-PAGE) gels. Proteins were transferred to nitrocellulose membranes (GE Healthcare UK Ltd., Buckinghamshire, Germany) using a Bio-Rad tank blotting apparatus (Bio-Rad). The protein-blotted membranes were probed with specific targeting primary antibodies, washed, and incubated with horseradish peroxidase-linked secondary antibodies. After the membranes were washed three times, the signals were detected with EzWestLumi One enhanced chemiluminescence solution (Atto Corporation, Tokyo, Japan) using a Fujifilm LAS-3000 (Fuji Photo, Tokyo, Japan).

## 5. Conclusions

We report for the first time that TTK extract functions as a potent inhibitor of PCOS associated with endocrine and metabolic abnormalities. The beneficial therapeutic effects of TTK extract on PCOS are mediated through inhibition of the ERK-CREB signaling pathway. TTK extract inhibits FOR-stimulated DHEA or testosterone via CYP17A1 and HSD3B2 and reduces the degree of ERK-CREB activation in NCI-H295R cells. Further, TTK extract treatment suppressed LH and testosterone in a letrozole-induced PCOS rat model. These results suggest that TTK extract could be developed and used to improve the treatment of PCOS resulting from excess androgen production.

## Abbreviations

TTK	*Tetragonia tetragonioides* (Pall.) Kuntze;
PCOS	Polycystic ovary syndrome;
NCI-H295R cells	NCI-H295R human adrenocortical cells;
DHEA	dehydroepiandrosterone;
FOR	forskolin;
CYP17A1	17α-hydroxylase/17,20-lyase;
HSD3B2	3β-hydroxysteroid dehydrogenase/Δ^5^-Δ^4^-isomerase type 2;
LH	luteinizing hormone;
E2	17β-estradiol;
ERK	extracellular signal-regulated kinase;
CREB	cAMP response element-binding protein
